# Physicochemical, Structural and Antioxidant Properties of Collagens from the Swim Bladder of Four Fish Species

**DOI:** 10.3390/md20090550

**Published:** 2022-08-26

**Authors:** Ye Dong, Zhiyuan Dai

**Affiliations:** Collaborative Innovation Center of Seafood Deep Processing, Key Laboratory of Aquatic Products Processing of Zhejiang Province, Institute of Seafood, Zhejiang Gongshang University, Hangzhou 310035, China

**Keywords:** swim bladder, pepsin-solubilized collagen, antioxidant activity, amino acids profile, structure

## Abstract

This study aimed to isolate and characterize pepsin-solubilized collagen (PSC) from marine and freshwater fish swim bladders. The physicochemical properties, protein pattern, amino acid composition, structure, thermal denaturation temperature, and antioxidant activity of PSC from four different swim bladder sources were investigated and compared. The results demonstrated that the four types of collagen extracted were all type I collagen. The yield of PSC extracted from grass carp (GCSB-PSC), bighead carp (BCSB-PSC), grouper (GSB-PSC), and monkfish swim bladders (MSB-PSC) were 38.98, 27.97, 18.16, and 10.35%, respectively. Compared to the other three PSCs, BCSB-PSC has the highest thermal denaturation temperature (38.60 °C). Based on FTIR spectroscopy and circular dichroism (CD) analysis, the extracted PSCs retained the triple helix and secondary structure well. Antioxidant studies showed that in the swim bladders of four species the swim bladder PSC could scavenge DPPH and ABTS radicals. Overall, swim bladders from marine and freshwater fish can be utilized as raw materials for collagen extraction, and the extracted collagen has potential commercial applications.

## 1. Introduction

Collagen is a large family of glycoprotein molecules which are the dominant protein components in connective tissue, accounting for about 30% of the body’s total protein [1]. Currently, approximately 29 types of collagen have been identified in various tissues; the most common being type I collagen [2]. The universal structural feature of collagen is a triple helix structure with 3 α-chains entwined into a triple helix. The α-chain is the basic unit of collagen and contains one or more polypeptide fragments Gly-X-Y, forming a triple helix structure with one or more non-triple helices [3]. Collagen has been widely used in the medical, cosmetic, leather, bone grafting, tissue engineering, and food industries owing to its weak antigenicity, degradability, good biocompatibility, and bioactivity [4]. On the other hand, collagen can be proteolyzed to produce collagen peptides that have bioactivities such as promoting wound healing, antioxidant, and reducing blood pressure [5,6,7]. Due to the growing demand for collagen, the production of collagen is growing and the market size of the collagen industry is expected to reach USD 6.63 billion by 2025 [8]. Aquatic fauna, including various vertebrate fish and invertebrates (sponges, sea cucumbers, jellyfish, etc.) are essential sources of collagen that have no religious restrictions and are free of diseases such as foot-and-mouth disease (FMD), transmissible spongiform encephalopathy (TSE), and bovine spongiform encephalopathy (BSE) [3]. Some collagen extracted from fish and fish processing byproducts, such as tilapia skin [4], bigeye tuna skin [9], and red drum fish scale [10], have been reported in recent years. Collagen extraction from these byproducts can help reduce the waste of protein resources, reduce environmental pollution, and realize the high-value utilization of low-value products. This utilization of waste products can enhance the economic value and social benefits of collagen extraction, and meet the market demand for natural, nutritious, and multifunctional products.

The swim bladder is one of the byproducts of fish processing and its main component is high-grade collagen, an excellent raw material for collagen extraction [11]. Except for some swim bladders used for food or medical applications, a large amount of swim bladders are discarded as waste, which wastes resources and pollutes the environment. Therefore, it is important to realize the efficient utilization of swim bladders. Currently, research on swim bladders has focused chiefly on the isolation and evaluation of bioactive peptides and the extraction of macromolecular collagen [11,12]. In the study of collagen extraction, Pal et al. [13] extracted type I collagen with a good triple helix structure and protofibril formation ability from the rohu swim bladder. Zhang et al. [14] extracted PSC from the Amur sturgeon swim bladder and observed that it has a good fibril-forming ability and thermal stability. It can be seen that collagen extracted from swim bladders has enormous potential for application in biomaterials. However, none of the studies have compared the differences in the properties of collagen extracted from marine and freshwater fish swim bladders. There is almost no information on collagen extracted from the swim bladders of monkfish and groupers. Therefore, clarifying the properties of swim bladder collagen from a variety of different sources is essential for subsequent studies regarding application and activity, as well as for the efficient use of marine and freshwater fish resources. The objective of this study was to extract PSC from the monkfish, grouper, bighead carp, and grass carp swim bladders, then characterize their physicochemical properties, structure, and further evaluate their antioxidant activity.

## 2. Results and Discussion

### 2.1. Yield

**Figure 1:**
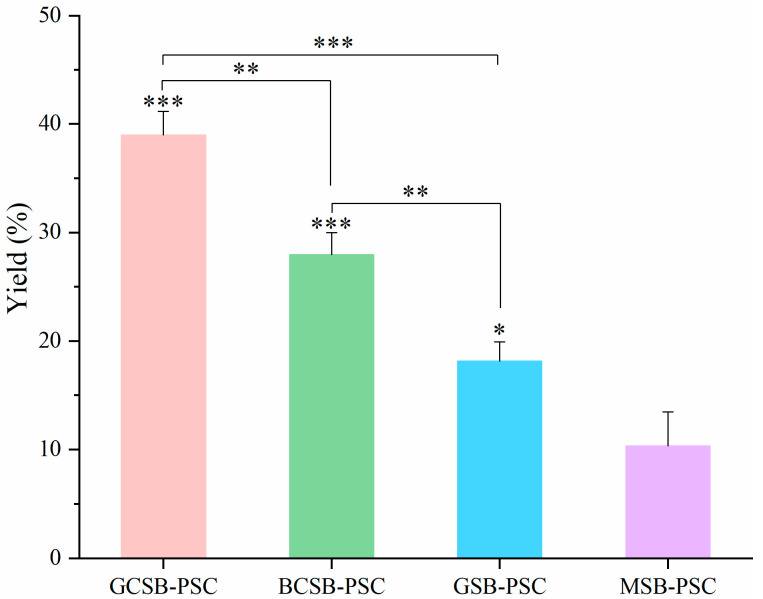
The yield of GCSB-PSC, BCSB-PSC, GSB-PSC, and MSB-PSC is based on dry-weight swim bladders. Significance is indicated by * (*, **, and *** indicate *p* ≤ 0.01, 0.001, and 0.0001, respectively).

As displayed in Figure 1, the yield of GCSB-PSC, BCSB-PSC, GSB-PSC, and MSB-PSC were 38.98, 27.97, 18.16, and 10.35%, respectively. There were significant differences in PSC yields from swim bladders of the four species (*p* ≤ 0.01). Higher yields of PSC were obtained from GCSB than from BCSB, GSB, and MSB. Additionally, GCSB-PSC yields were higher than that of PSC extracted from yellowfin tuna swim bladders (12.10%) [15] and tilapia skin (20.03%) [16]. The yield of GCSB-PSC was comparable to the collagen yield extracted from grass carp swim bladders (39.2%) by Li et al. [11]. Le et al. found that the yield of collagen obtained from the fish skin from different species was different [17]. The discrepancies in collagen yield might be attributed to biological conditions, preparation methods, extraction source, differences in collagen structural fiber cross-linking, and the state of byproducts derived from the process [3,18]. Meng et al. [19] found that alkaline pretreatment of sturgeon skin and notochord improved the efficiency of collagen extraction but reduced collagen yield. In this study, the different yields of the four swim bladder PSCs might be due to the different thermal sensitivity of collagen in fish species from different habitats, and the differences in the swim bladder matrix and alignment distribution of the swim bladder components. The compactness of the swim bladder matrix varies in different fish species, which may affect the degree of acetic acid penetration into the swim bladder matrix and the degree of collagen loss during the alkali-pretreatment; thereby affecting the yield of collagen. Additionally, the collagen content of the raw material may also influence the yield. Further studies are needed on how to improve the yield of collagen, for example, by optimizing the extraction conditions (acetic acid concentration, solvent/solids ratio, extraction time, etc.), pretreatment methods, and the use of collagenase for extraction, as the matrix composition of different fish can vary.

### 2.2. Protein Pattern

**Figure 2:**
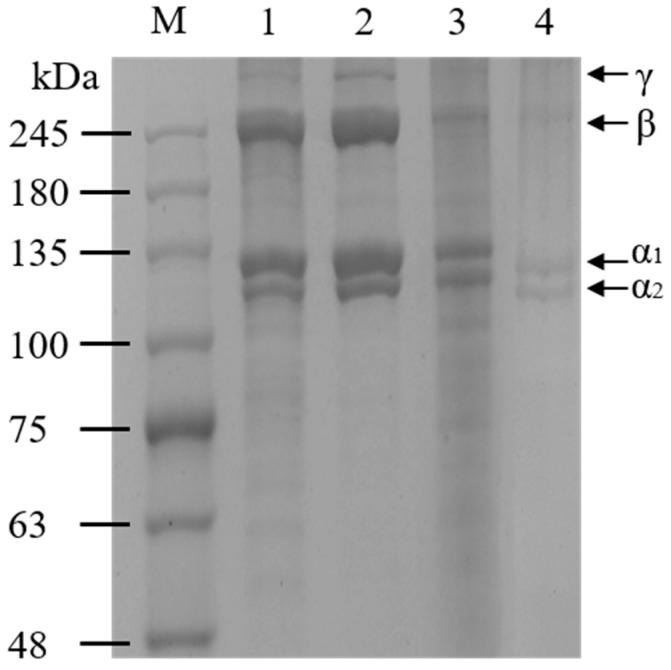
SDS-PAGE patterns of PSC from four species’ swim bladders. M: molecular weight markers; 1: GCSB-PSC; 2: BCSB-PSC; 3: GSB-PSC; 4: MSB-PSC.

As illustrated in Figure 2, GCSB-PSC, BCSB-PSC, GSB-PSC, and MSB-PSC exhibited similar protein patterns. They all contain α1- and α2-chains in an approximate ratio of 2:1, consistent with the characteristics of type I collagen [11]. The molecular weights of α1-, α2-, and β-chain were approximately 133, 123, and 255 kDa, respectively. Similar protein patterns in collagen from catfish waste were reported by Abbas et al. [20]. In addition to α-chain, high molecular weight components such as β-chain and γ-chain are also present in PSC, which are similar to collagen derived from the swim bladders of miiuy croaker [2], rohu [13], and yellowfin tuna [15]. β-chain presence suggests that the extracted collagen contains intermolecular interactions [20]. Compared with MSB-PSC and GSB-PSC, the intensity of the high molecular weight bands, i.e., β and γ chains, was higher in GCSB-PSC and BCSB-PSC, indicating a higher number of crosslinks in GCSB-PSC and BCSB-PSC. Cross-linking is among the factors that make collagen extraction difficult. The degree of cross-linking of MSB-PSC was low as evidenced by the electropherogram, but the yield was also low as shown in Section 2.1. Therefore, cross-linking may not be the main factor affecting the collagen yield of the four swim bladders in this study.

### 2.3. Amino Acid Composition

**Figure 3:**
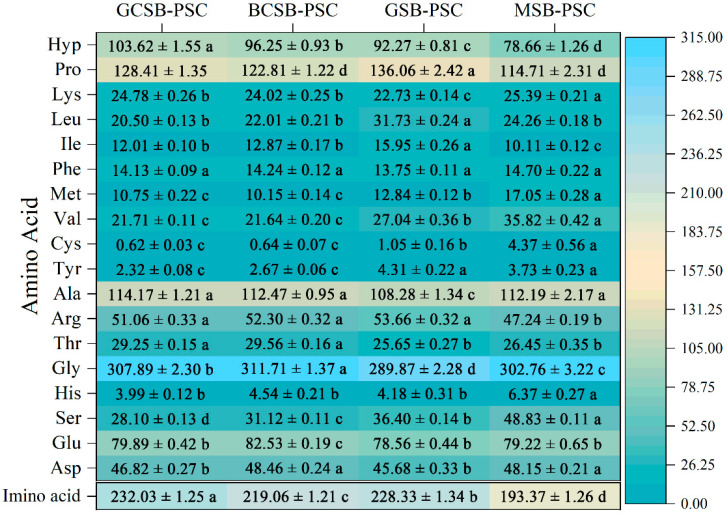
Heatmap of the amino acid composition of GCSB-PSC, BCSB-PSC, GSB-PSC, and MSB-PSC (residues/1000 total amino acid residues). The gradual change of the color scale value from green-yellow-blue indicates the gradual increase in amino acid residues. Different letters denote the significant difference (*p* < 0.05).

A heat map of the amino acid composition of the PSCs is exhibited in Figure 3. Glycine (289.87–311.71 residues/1000 residues) has the highest proportion of total amino acid residues (approximately one-third of the total residues), followed by proline (114.71–136.06 residues/1000 residues), alanine (108.28–114.17 residues/1000 residues), and hydroxyproline (78.66–103.62 residues/1000 residues). The structure of collagen is characterized by a Gly-X-Y repeat sequence, dominated by proline and hydroxyproline at the X and Y positions [21]. These two amino acids are characteristic amino acids of collagen, which are typically found only in collagen and are very rare in other proteins. Therefore, the amino acid composition of swim bladder collagen has a higher content of glycine, proline, and hydroxyproline. From the heat map, it can be seen that the PSC of the four swim bladders exhibited slight differences in amino acid composition. The glycine content was highest in BCSB-PSC, proline was highest in GSB-PSC, and hydroxyproline was highest in GCSB-PSC. Among the swim bladders, tyrosine, histidine, and cysteine were at extremely low levels. In GCSB-PSC and BCSB-PSC the cysteine content was negligible. Amino acid content and composition affect the properties of collagen from different swim bladder sources. For example, glycine facilitates the tight binding of the α-chains in the collagen triple helix to form a superhelix [22].

The amount of imino acid (hydroxyproline and proline) in collagen is vital for the stability of the triple helix structure of collagen and contributes to the maintenance of collagen’s thermal stability [23]. The pyrrolidine ring of imino acids could restrict the secondary structure changes of the polypeptide chain, and therefore, contribute to the maintenance of the triple helix structure of collagen [24]. Furthermore, hydroxyproline stabilizes the triple helix structure through interchain hydrogen bonds formed by hydroxyl groups [25]. The imino acid contents of GCSB-PSC, BCSB-PSC, GSB-PSC, and MSB-PSC were 232.03, 219.06, 228.33, and 193.37 residues/1000 residues, respectively, which were higher than those reported for the swim bladders of yellowfin tuna (169 residues/1000 residues) [15], giant croaker (187.2 residues/1000 residues) [26], and grass carp (175 residues/1000 residues) [11]. The highest imino acid content in this study was in GCSB-PSC and the lowest imino acid content was found in MSB-PSC. The change in collagen imino acid content of different swim bladders was related to the habitat temperature of the material fish. In general, the collagen obtained from warm-water fish had a higher imino acid content than that obtained from cold-water fish [27]. Grass carp, bighead carp, and groupers are warm-water fish, while monkfish are cold-water fish; thus, its collagen imino acid content is relatively low. The difference in the collagen imino acid content of the swim bladders of marine and freshwater fish presumably reflects changes in thermal denaturation temperature and triple helix strength.

### 2.4. FTIR Spectroscopy

**Figure 4:**
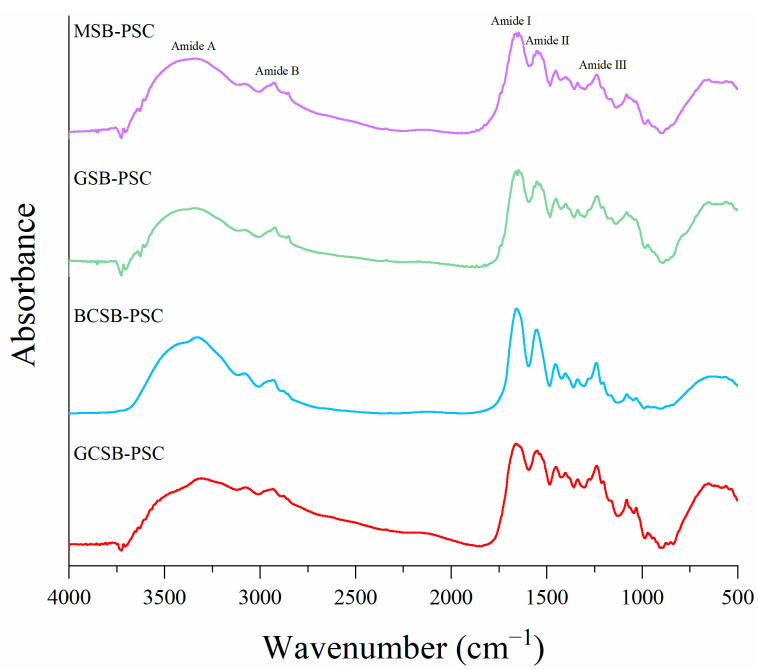
FTIR spectra of GCSB-PSC, BCSB-PSC, GSB-PSC, and MSB-PSC.

Figure 4 displays the FTIR spectra of the PSC of the four swim bladders, which exhibit similar spectral properties and all contain characteristic bands of amides A, B, I, II, and III. Among them, the amide A band (3400–3440 cm^−1^) is generally correlated with the hydrogen bonding and N-H stretching vibrations on peptides within collagen. Whilst the N-H group participated in the hydrogen bond formation, the peak of the amide A band will shift to a relatively low wavenumber of about 3300 cm^−1^ [28]. The amide A bands of GCSB-PSC, BCSB-PSC, GSB-PSC, and MSB-PSC were located near 3307.90, 3331.38, 3335.58, and 3335.91 cm^−1^ wavelengths, respectively, demonstrating that the N-H groups on the peptides within the PSC were involved in the forming of hydrogen bonds. GCSB-PSC had the lowest wavelengths in the amide A band, indicating the hydrogen bonding intensity of GCSB-PSC was greater than that of the other three PSCs. The amide B bands of PSC from GCSB, BCSB, GSB, and MSB were noticed at 2932.47, 2932.73, 2923.40, and 2927.13 cm^−1^ wavenumbers, respectively, which were associated with the asymmetric stretching of CH_2_ [29]. The amide I, amide II, and amide III band wavenumbers were correlated directly with the conformation of collagen. The amide I band, located at 1600–1700 cm^−1^, mainly involves C=O stretching vibrations and could serve as a sensitive marker for the analysis of collagen secondary structures. Wu et al. [18] reported that the amide I band shifts to a lower wavelength, which is associated with a reduction in molecular order, and that the removal of some terminal peptides by pepsin during the extraction of PSC may lead to a loss of active amino acids. Amide I bands of GCSB-PSC, BCSB-PSC, GSB-PSC, and MSB-PSC were observed at 1658.23, 1657.72, 1646.82, and 1647.06 cm^−1^, respectively. The amide I band wavenumbers of GSB-PSC and MSB-PSC were lower than those of GCSB-PSC and BCSB-PSC, indicating more reduction in molecular order in GSB-PSC and MSB-PSC. The amide II bands (1550–1600 cm^−1^) of the four swim bladder collagens were found in the range of 1548–1554 cm^−1^, with a decrease in the wavenumber suggesting the formation of hydrogen bonds [3]. The decrease in the wavenumber of the collagen amide II band of the four swim bladders indicates the formation of hydrogen bonds, which are conducive to the stabilization of the triple helix structure. The absorption peak (1235–1240 cm^−1^) of the amide III band involves collagen intermolecular interactions and the triple helix structure, which also results from the wobble vibration of the CH_2_ group of glycine and proline [30]. Amide III bands were found at 1238.70, 1240.08, 1237.59, and 1238.71 cm^−1^ for GCSB-PSC, BCSB-PSC, GSB-PSC, and MSB-PSC, respectively, suggesting that they have helical structures [26]. Furthermore, the absorption peaks of GCSB-PSC, BCSB-PSC, GSB-PSC, and MSB-PSC were found at 1451.15, 1453.52, 1451.40, and 1451.70 cm^−1^, respectively, which are pyrrolidine ring vibrations of imino acids. The closer the intensity ratio between the wavenumber of the amide III band and the wavenumber around 1450 cm^−1^ is to one, the more the triple helix structure of the extracted collagen is intact [3]. The ratios of the amide III bands of GCSB-PSC, BCSB-PSC, GSB-PSC, and MSB-PSC to 1451.15, 1453.52, 1451.40, and 1451.70 cm^−1^ were 1.01, 1.02, 1.03, and 0.98, respectively, demonstrating that they maintained a better triple helix structure. The results showed that the FTIR spectra of the swim bladders’ PSC had a distinct collagen feature, similar to the skin of blacktip reef sharks [31] and the swim bladder of gulf corvina [32].

**Figure 5:**
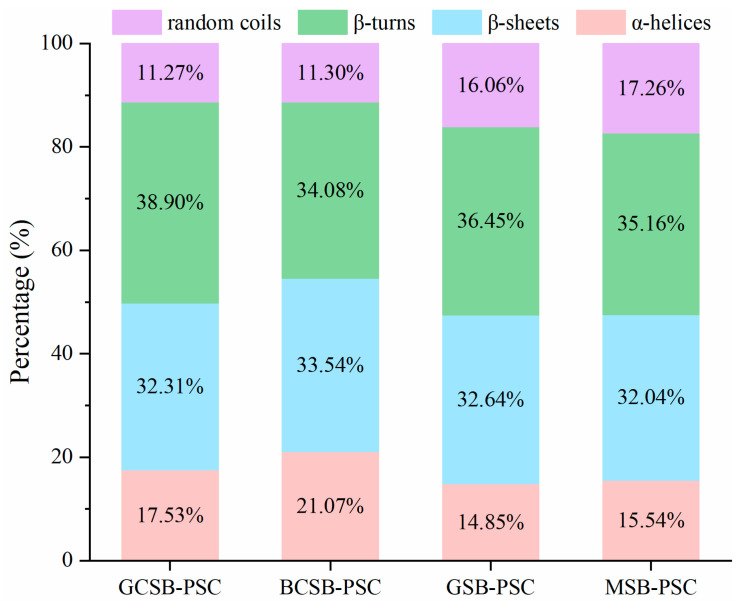
The relative contents of the secondary structure of GCSB-PSC, BCSB-PSC, GSB-PSC, and MSB-PSC.

The Gaussian peak fitting algorithm was used to determine the secondary structure pattern of collagen, and the results are shown in Figure 5. The secondary structure of collagen mainly consists of β-sheets, β-turns, α-helices, and random coils. From the figure it is clear that GCSB-PSC, BCSB-PSC, GSB-PSC, and MSB-PSC contain 32.31, 33.54, 32.64, and 32.04% of β-sheets, 38.90, 34.08 36.45, and 35.16% of β-turns, 17.53, 21.07, 14.85, and 15.54% of α-helices, 11.27, 11.30, 16.06, and 17.26% of random coils, respectively. Of the α-helices with tight structures and high stability, BCSB-PSC had the highest α-helix content and probably had higher stability among the four PSCs. The β-sheet has fewer intermolecular hydrogen bonds; therefore, the secondary structure is not as stable as that of the α-helix. β-turns and random coils are associated with protein unfolding, dissociation, and rearrangement [11]. The α-helix and β-sheet of the four swim bladder PSCs were higher than that of the grass carp skin PSC (α-helix: 9.26%, β-sheet: 13.35%) reported by Zhu et al. [33], demonstrating that the secondary structures of the swim bladder PSCs extracted in this study were more tightly connected.

### 2.5. UV Absorption Spectrum

**Figure 6:**
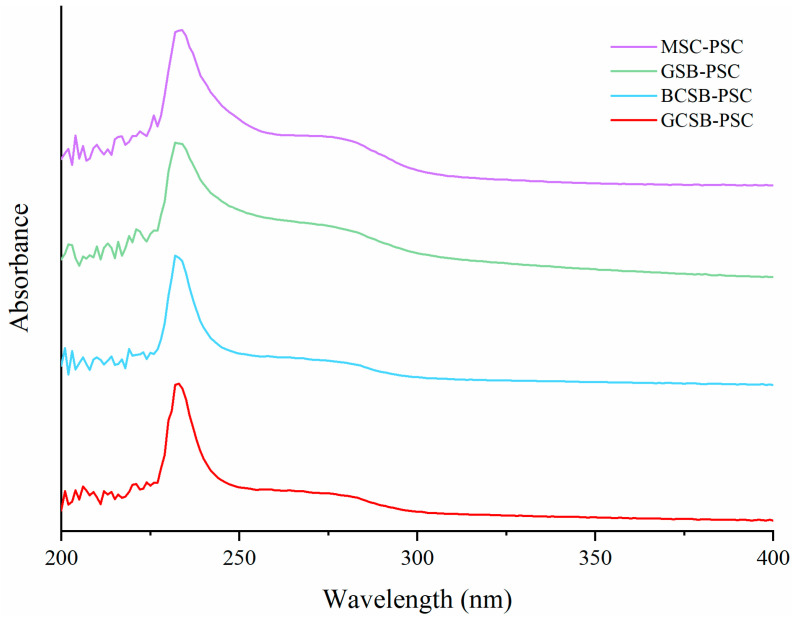
UV spectra of GCSB-PSC, BCSB-PSC, GSB-PSC, and MSB-PSC.

The carbonyl, carboxyl, and amide groups in the collagen peptide chain are all chromogenic groups, which display strong UV absorption between wavelengths of 200–400 nm. Collagen triple helix structure has a maximum absorption peak at approximately 230 nm. As shown in Figure 6, the PSC of the four swim bladders has a maximum absorption peak at about 234 nm generated by the triple helix structure of collagen [34]. Ge et al. [31] also observed a maximum absorption peak at 234 nm with collagen extracted from blacktip reef shark skin. Generally, histidine, cysteine, and aromatic amino acids have UV absorption of around 250 to 280 nm, whereas, the content of these amino acids in pure collagen is very low. The swim bladder PSC of marine and freshwater fish extracted in this study showed no significant UV absorption in the range of 250–280 nm, indicating a low content of these amino acids, which is consistent with the amino acid results and further demonstrates the high purity of the extracted collagen.

### 2.6. CD Analysis

**Figure 7:**
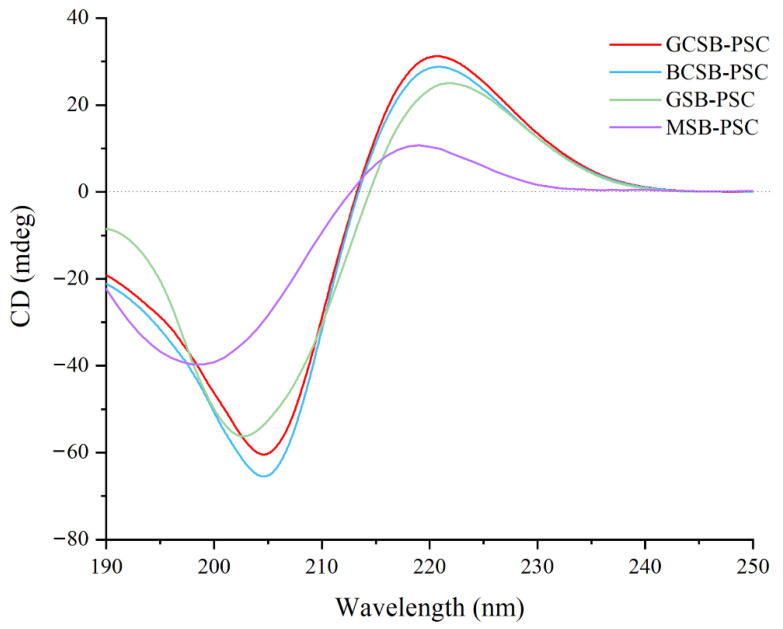
CD spectra of GCSB-PSC, BCSB-PSC, GSB-PSC, and MSB-PSC.

CD is an efficient approach to confirming the structural integrity of the triple helix. The collagen triple helix structure has a positive absorption peak at approximately 220 nm and a negative absorption peak at approximately 200 nm [3]. The ratio of the absolute value of the positive peak intensity to the negative peak intensity (Rpn) has been applied to establish the presence of a triple helix conformation [35]. If the Rpn value is less than one, the extracted collagen can be judged to have a triple helix structure. As shown in Figure 7, GCSB-PSC, BCSB-PSC, GSB-PSC, and MSB-PSC have positive peaks at 220, 220, 221, and 219 nm and negative peaks at 204, 204, 202, and 199 nm, respectively. Their Rpn values were 0.51, 0.43, 0.44, and 0.27, respectively, all of which were less than one, indicating that the extracted PSCs of the four fish bladders retained the natural triple helix structure.

### 2.7. DSC Analysis

**Figure 8:**
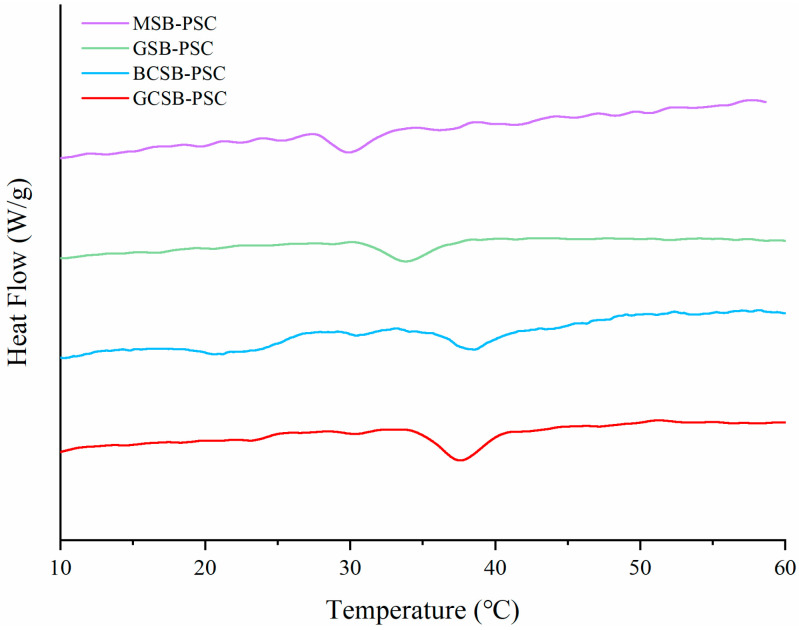
DSC curves of GCSB-PSC, BCSB-PSC, GSB-PSC, and MSB-PSC.

Figure 8 shows the DSC heat flow diagrams of four fish bladder collagens. The highest thermal denaturation temperature was observed for BCSB-PSC (38.60 °C), followed by GCSB-PSC (37.61 °C). The thermal denaturation temperature of GSB-PSC (33.84 °C) was similar to that of giant croaker swim bladder collagen (33.8 °C) [26]. Lower thermal denaturation temperatures were observed in MSB-PSC (29.85 °C). The thermal denaturation temperatures of BCSB-PSC, GCSB-PSC, and GSB-PSC were higher than that of the swim bladder collagen of Atlantic cod (29.6 °C) [36], as well as the scale-PSC (31.6 °C) and bone-PSC (32.3 °C) of bigeye tuna [37]. The thermal stability of collagen is largely related to the pyrrolidine ring of the imino acid, and this structure plays a vital role in the stability of the triple helix structure [13]. That means the thermal denaturation temperature of collagen is positively associated with the content of imino acids [27]. The thermal denaturation temperatures of the BCSB-PSC and GCSB-PSC were higher than that of the collagen from the grass carp swim bladder reported by Li et al. (34.3 °C), which may be attributed to the higher imino acid contents of BCSB-PSC and GCSB-PSC than those reported by Li et al. [11]. However, the imino acid content of GSB-PSC was higher than that of BCSB-PSC, but its thermal denaturation temperature was lower than that of BCSB-PSC. Nagai et al. [38] demonstrated that the hydroxyl structure in hydroxyproline contributes to the formation of internal hydrogen bond bonding and can enhance the stability of the triple helix structure; therefore, there was a positive correlation between the proline hydroxylation degree and the thermal denaturation temperature of collagen. The thermal stability of collagen is also related to the body temperature of the fish and the ambient temperature in which it lives [13]. From the amino acid results, the total imino acid content of GSB-PSC was higher than that of BCSB-PSC, while its hydroxyproline content was lower than that of BCSB-PSC. Secondly, the optimal growth temperature of grouper is 22–28 °C, which is lower than the survival environment temperature of bighead carp (25–30 °C). Therefore, the thermal denaturation temperature of GSB-PSC was lower than that of BCSB-PSC. Generally, cold-water fish collagen is less thermally stable than warm-water fish collagen [27,39]. Akita et al. compared the thermal stability of collagen from six species of warm-water fish and five species of cold-water fish and found that warm-water fish had higher thermal stability [27]. Monkfish is a cold-water fish, resulting in a low denaturation temperature of the extracted collagen. This study suggested that GCSB-PSC and BCSB-PSC have a higher thermal denaturation temperature than MSB-PSC. The thermal stability of PSC mainly depends on the content of hydroxyproline as well as the environmental temperature in which the fish live, and the thermal stability of warm-water fish PSC was higher than cold-water fish.

### 2.8. Antioxidant Activity

**Figure 9:**
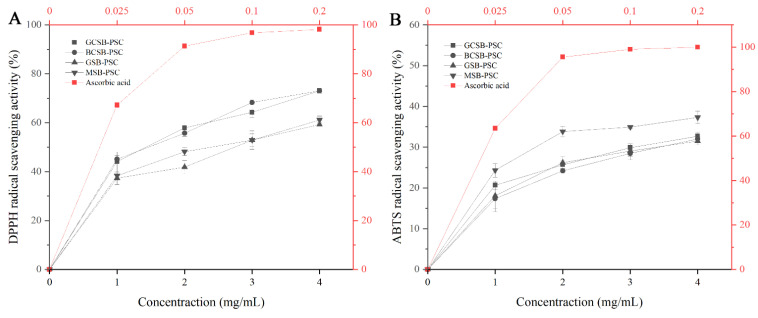
DPPH radical (**A**) and ABTS radical (**B**) scavenging activities of GCSB-PSC, BCSB-PSC, GSB-PSC, and MSB-PSC.

It is well known that collagen plays a vital role in wound healing and skin cell renewal. The regulation of oxidative stress and inflammation plays a vital role in the process of wound healing [40]. Reactive oxygen species are the dominant cause of oxidative stress. Therefore, it is important to study the antioxidant properties of collagen. In this study, the antioxidant activity of four fish bladder PSCs was evaluated. As shown in Figure 9, PSC (1–4 mg/mL) obtained from four fish bladders could scavenge DPPH and ABTS free radicals. Among them, MSB-PSC exhibited higher ABTS radical scavenging activity than the other three PSCs. GCSB-PSC and BCSB-PSC showed similar scavenging activity for DPPH radicals and were better than the GSB-PSC and MSB-PSC. The scavenging activity of DPPH radicals by the PSC in this study was higher than that of *Lophius litulo* skin PSC [41]. The PSC seems to scavenge DPPH radicals better than ABTS radicals. At a concentration of 3 mg/mL, the four PSCs all showed over 50% scavenging activity for DPPH radicals, while the scavenging activity for ABTS radicals was only 29–35%. In the literature the antioxidant activity of collagen extracted from swim bladders has been reported. For instance, Zhao et al. found that ASC and PSC extracted from the swim bladders of miiuy croaker were able to scavenge free radicals such as DPPH and superoxide anion [2]. In addition, collagen extracted from a giant croaker swim bladder also showed antioxidant activity with scavenging DPPH, hydroxyl, superoxide anion, and ATBS radical free radicals [26]. Our study results are consistent with these previous research findings. However, the antioxidant activity of the collagen extracted in this study was relatively low compared to ascorbic acid. It should be subsequently enhanced by the addition of other functional components such as chitosan before incorporating it into wound healing applications [42,43].

## 3. Materials and Methods

### 3.1. Materials

Grass carp (*Ctenopharyngodon idella*), Monkfish (*Lophiiformes*), Grouper (*Epinephelus*) and Bighead carp (*Aristichthys nobilis*) were obtained from the aquatic market in Hangzhou, Zhejiang, China. Swim bladders were taken immediately after fish slaughter, packed in plastic bags, and frozen, then transported on ice to the laboratory and immediately stored at −60 °C until use. Acetic acid, ethanol, sodium hydroxide, and sodium chloride were of analytical grade and purchased from Sinopharm Chemical Reagent Co., Ltd. (Shanghai, China). Rainbow 245 plus protein marker, pepsin 1:3000 (porcine stomach mucosa, 3000–3500 NFU/g), and SDS-PAGE loading buffer 5× (with DTT) were purchased from Solarbio Science & Technology Co., Ltd. (Beijing, China). All the other reagents used were at an analytical grade.

### 3.2. Preparation of Pepsin-Soluble Collagen (PSC)

Fresh swim bladders were cleaned with tap water to remove blood stains and impurities and cut into small pieces (1 cm × 1 cm × 1 mm), then mixed with NaOH (0.1 M) for 24 h (1:20 *w/v*, the fresh solution was replaced every 8 h) at 4 °C. After that, the swim bladders were washed with prechilled purified water with a neutral pH then treated with 10% ethanol for 24 h to remove fat, followed by washing with prechilled purified water to remove residual ethanol and freeze-drying, then stored at −30 °C.

The pretreated swim bladder was soaked in 0.5 M acetic acid solution (1:20 *w*/*v*, containing 0.05% pepsin) and extracted through continuous stirring at 4 °C for 48 h. The extracted solution was centrifuged (4 °C) at 1000× *g* for 20 min, and the residue was further extracted (48 h). The supernatant was mixed with the earlier extraction to obtain the crude extract of PSC. Then NaCl solution was slowly added to the crude extract to make the final concentration of 1.0 M to precipitate the PSC. The precipitate was collected through centrifugation (10,000× *g* for 20 min at 4 °C), then redissolved with 0.5 M acetic acid and dialyzed against using dialysis membranes (MWCO: 100 kDa). 50 mL of each sample was first dialyzed in 1500 mL of 0.1 M acetic acid for 24 h, followed by 48 h in distilled water. Lastly, the PSC was freeze-dried, and the dry PSC was stored at −60 °C. The PSC obtained from the swim bladders of monkfish, grouper, grass carp, and bighead carp were named MSB-PSC, GSB-PSC, GCSB-PSC, and BCSB-PSC, respectively. 

The PSC yield was calculated using the following formula:

Yield (%) = (Weight of freeze-dried collagen (g)/Weight of dry swim bladder (g)) × 100(1)

### 3.3. Protein Pattern

The protein patterns of purified PSC were analyzed with the aid of sodium dodecyl sulfate-polyacrylamide gel electrophoresis (SDS-PAGE) [11]. PSC was dissolved in 0.5 M acetic acid (1 mg/mL), mixed with 5× SDS-PAGE loading buffer (4:1), and boiled for 5 min. Then 10 µL was loaded onto 7% separating gel and 4% stacking gel for electrophoresis. The gels were stained with 0.25% Coomasie Brilliant Blue R-250 solution for 30 min, followed by 10% acetic acid and 10% methanol mixed solutions until the protein bands were clear.

### 3.4. Fourier Transform Infrared Spectroscopy (FTIR)

PSC (1 mg) was mixed with potassium bromide (100 mg), ground evenly, pressed into thin slices, and detected with a Nicolet iS5 FTIR spectrometer (Waltham, MA, USA). The scanning range was 400–4000 cm^−1^, and the resolution was 4 cm^−1^ [11].

### 3.5. Ultraviolet (UV) Absorption

The PSC was dissolved with 0.5 M acetic acid solution at a concentration of 0.5 mg/mL and scanned at 190–400 nm (scan interval 1.0 nm) using a full-wavelength UV spectrophotometer (Thermo Scientific Evolution 60S, Waltham, MA, USA) with 0.5 M acetic acid as a blank for baseline calibration.

### 3.6. Circular Dichroism (CD)

The PSC was dissolved in 0.5 M acetic acid and placed in a quartz cell (1 mm). After that, measurements were performed with a CD spectrometer (J-1500, JASCO, Tokyo, Japan) at a scan rate of 10 nm/min at 190–250 nm and 0.5 M acetic acid was used as the blank [29].

### 3.7. Differential Scanning Calorimetry (DSC)

DSC was used for measuring the thermal denaturation temperature of collagen. The lyophilized PSC was rehydrated in deionized water (1:40 *w*/*v*) for 48 h at 4 °C according to the method of Ali et al. [44]. A 5 mg sample was weighed into an aluminum container, sealed, then analyzed under N_2_ atmosphere at a heating rate of 2 °C/min in the range of 0–70 °C and an empty aluminum pan as a reference. The maximum transition temperature (*T_max_*) was determined by observing the endothermic peaks in the DSC thermogram.

### 3.8. Amino Acid Profile

The PSCs were hydrolyzed with 6 M hydrochloric acid in a sealed tube at 110 °C under vacuum for 22 h. The amino acid composition of PSC was measured using the method of Dong et al. [45] using an Agilent 1100 series HPLC system (Agilent Technologies, Inc., Santa Clara, CA, USA) equipped with a C18 ODS Hypersil column (4.6 mm 250 mm i.d., 5 m particle size; Agilent Technologies, Inc.) for the analysis. Hydroxyproline was detected at 262 nm and the detection wavelength of the other amino acids was 338 nm.

### 3.9. Antioxidant Activity

Antioxidant activity of PSC was expressed by measuring the DPPH and ABTS radical scavenging activity.

The DPPH radical scavenging activity of PSC was determined according to Devita et al. [9] with some modifications. 100 μL PSC (dissolved in 0.5 M acetic acid) was mixed with DPPH solution (100 μL). The mixture was incubated (30 min) in the dark and then the absorbance was measured at 517 nm. The DPPH radical scavenging rate was calculated according to the following formula:

DPPH radical scavenging activity (%) = [1 − (A_1_ − A_2_)/A_0_] × 100 (2)

where A_1_: the absorbance of PSC; A_2_: the absorbance of sample blank; A_0_: the absorbance of the control group without PSC.

The ABTS radical scavenging activity of the PSC was carried out according to Dong et al. with some modifications [46]. PSC (100 μL) was mixed with ABTS diluted solution (100 μL). Then the sample mixtures were incubated (10 min) at room temperature and then the absorption was monitored at 734 nm. The ABTS radical scavenging activity was calculated as follows:

ABTS radical scavenging activity (%) = [1 − (A_1_ − A_2_)/A_0_] × 100 (3)

where A_1_: the absorbance of PSC sample; A_2_: the absorbance of sample blank; A_0_: the absorbance of the control without PSC.

### 3.10. Statistical Analysis

Data were presented as the mean ± standard deviation (SD) and calculated using SPSS software version 21.0 (SPSS Inc., Chicago, IL, USA) for Duncan’s multiple range tests. All analyses were performed in triplicate (n = 3), using one-way ANOVA, and values of *p* < 0.05 were considered statistically significant.

## 4. Conclusions

In this study, PSC was successfully extracted from the swim bladders of marine and freshwater fish. The properties of collagen obtained from different swim bladders are slightly different. The yield of GCSB-PSC was significantly higher than that of BCSB-PSC, followed by GSB-PSC, and MSCB-PSC. The thermal stability of BCSB-PSC was higher than that of GCSB-PSC, followed by GSB-PSC. MSCB-PSC had the lowest thermal stability, which could be attributed to its lower imino acid content and the fact that it is a cold-water fish. All PSCs were described as type I collagen, they maintained a good triple helix structure, and they had antioxidant activity. The results of this study provide beneficial information for the preparation of collagen from different sources of swim bladders for applications in biomaterial manufacturing and the food industry. These findings also contribute to the reduction of protein resource waste in fish processing byproducts.

## Data Availability

Not applicable.
